# FMI-based co-simulation method and test verification for tractor power-shift transmission

**DOI:** 10.1371/journal.pone.0263838

**Published:** 2022-02-11

**Authors:** Yiwei Wu, Yawei Mao, Liyou Xu

**Affiliations:** College of Vehicle and Traffic Engineering, Henan University of Science and Technology, Luoyang, Henan, China; Tongji University, CHINA

## Abstract

The tractor power-shift transmission (PST) research and development is a design process that incorporates many disciplines such as mechanical, control, and electronics. Modeling and simulation are typically dependent on various commercial tools for each discipline, making simulation, integration, and verification of system-level models problematic. Aiming at this, we propose a PST multi-domain co-simulation method based on the functional mock-up interface (FMI) standard, analyze the FMI-based simulation mechanism and the PST simulation system logical structure, and established the PST mechanical system model, control system model, tractor engine model, and tractor dynamic model. Based on FMI, these models are integrated into a PST co-simulation model. The starting speed, final drive half shaft speed and torque were simulated and tested. Among them, the simulation and the test starting time are 2.7s and 2.8s respectively, and the two speed curves are consistent; the simulation and the test final drive half shaft torque are approximately equal with a value of 1.5kN·m; the average Theil’s inequality coefficients (TIC) value of the simulation and the test final drive half shaft speed is 0.1375, which is less than 0.25. The results show that the simulation and the test results are consistent, the PST co-simulation model is accurate, and the co-simulation method is feasible, which can improve the efficiency of tractor PST system development.

## Introduction

The tractor power-shift transmission (PST) is a complex product that integrates multidisciplinary fields such as mechanics, control, and electronics [1]. PST is made up of three components: power shift transmission, central transmission, and final transmission. The essential component of PST is power shift transmission. It employs the transmission control unit (TCU) as its central processing unit to precisely control one or more pairs of shift clutches to disengage or engage, allowing for automatic gear shifting without causing tractor power to be interrupted when under load [2]. PST is frequently utilized in high-power tractors to improve dynamic performance, economy, comfort, safety, and efficiency [3]. At present, the commonly used individual simulation methods of PST subsystems in different fields have been unable to meet the need of PST product design. The co-simulation technology can solve the problems of difficult model interaction and poor model integration in the modeling and simulation of various disciplines [4–6], effectively shorten the model development and maintenance cycle, and improve the PST system development efficiency.

By establishing data interfaces between different tools, co-simulation enables real-time data exchange between various subsystems. As a result, engineers can combine several separate virtual models into one to achieve diverse modeling and simulation. However, there are still significant challenges in model integration in practice.

The MODELISAR project [7] has created a standard functional mock-up interface (FMI) for co-simulation that does not rely on tools [8]. It specifies clear specifications as well as an application programming interface (API) for integrating simulation components [9]. FMI is primarily involved in automotive design and simulation, and has already achieved industrial applications [10]. However, the research of this technology in the field of tractor PST product development has so far been rarely noticed.

Scholars have utilized different methods to study PST. The mathematical tools were used to establish the dynamic models of PST, by which the performance of transmission technology was predicted and evaluated [11–15]. Different software tools were used to model and simulate the functional components, control and hydraulic system of the tractor transmission system. For example, the MATLAB/Simulink and AMESim software were applied to establish the PST control and hydraulic model, and the function of the PST control system was studied [16,17], which can efficiently and quickly analyze the advantages and disadvantages of the transmission system control strategy, and reduce production costs. In addition, there is a research on the control function analysis of power shift tractor transmission system using Dymola software based on the Modelica language [18]. This method applies the visual modeling technology to the model establishment, making the modeling and simulation process more intuitive and user-friendly. However, these studies were limited to a certain aspect and not involved in the tractor PST co-simulation field. As a result, the complexity of the whole problem will be reduced, which will inevitably lead to the loss of system integrity and correlation, thereby reducing the confidence of the simulation results. And in most of the above modeling and simulation processes, there are problems such as insufficient compatibility of simulation tools and poor model integration, resulting in low model accuracy and dynamic characteristics far from the real thing. With the in-depth research of the co-simulation technology, the co-simulation and verification of the multi-domain modeling simulation environment and other simulation environments based on the Modelica language were carried out [19–21]. The effectiveness of multi-source heterogeneous model co-simulation based on the FMI standard was verified [22–26]. The problem of sharing various professional modeling software models is solved, and the scalability of the integrated simulation platform is improved, but there is a lack of research and application in the field of agricultural tractors.

To meet the current multi-domain modeling and simulation requirements of tractor PST, this paper proposes the co-simulation method based on FMI standard to study the PST system and establishes the co-simulation model of the PST system. The model accuracy and the method feasibility are verified with the combination of simulation and test.

The remaining part of the paper proceeds as follows: it starts by analyzing the FMI-based simulation mechanism and the PST simulation system logical structure. It will then go on to the PST co-simulation model establishment. The fourth section carries out the model verification. And the final section provides a concise summary of the key findings.

## Methodology

With the rapid development of simulation technology, single-disciplinary field simulation is gradually developing in the direction of multi-disciplinary field co-simulation. Simulation tools such as MATLAB/Simulink, Adams, and AMESim have been widely used in multi-disciplinary co-simulation [27–30]. These simulation tools provide a one-to-one interface for co-simulation, but this co-simulation method is only suitable for specific tools, and the interface is not universal, making multi-disciplinary co-simulation based on a unified modeling specification difficult to fully execute.

There are primarily two types of methodologies for integrating complex system simulation models currently available. One way is based on a unified interface, which implements information transfer across different tools via a unified interface while leaving the internal implementation process relatively unrestricted. For example, the method based on high level architecture (HLA) [31], however HLA is still incomplete in several areas, making it impossible to significantly improve the operational efficiency of run time infrastructure (RTI) [32], and RTI compatibility between manufacturers is weak. The other way is based on a common language, but it is not yet mature, and separate models must be constructed independently to suit the actual modeling and simulation requirements.

Taking into account variables such as platform scalability, simulation equipment cost, and operation ease, the use of common simulation model packaging interface standards to establish a collaborative simulation platform can partially overcome the following shortcomings: commercial tools have high requirements for software and hardware, the program’s non-open source nature limits the platform’s scalability, and simulators find it difficult to gain additional control over the simulation process.

**Fig 1:**
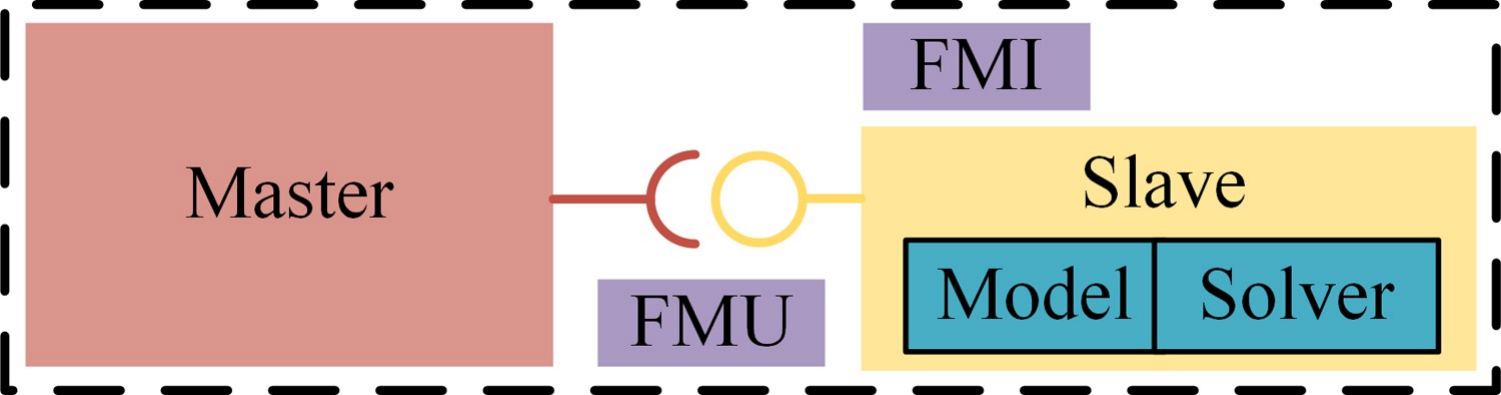
FMI-based co-simulation mechanism.

The FMI standard is a general third-party interface standard [33]. It not only specifies the way of data definition and preservation but also defines the interface of all externally accessible functions. It does not rely on the unique interface form of any tool. There is no need to develop a large number of software customization interfaces for co-simulation of models like traditional methods. It standardizes the model and data structure, and interface format, and provides effective support for solving the problems of poor model reuse, difficult interaction between models of various disciplines, and difficulty in simultaneous co-simulation of different tools [34]. Fig 1 shows the mechanism of FMI-based co-simulation mode. The model component functional mock-up unit (FMU) is derived from the slave program that contains the model and the solver. The master program calls FMU through the interface provided by the FMI standard to achieve co-simulation be-tween different simulation tools [35].

**Fig 2:**
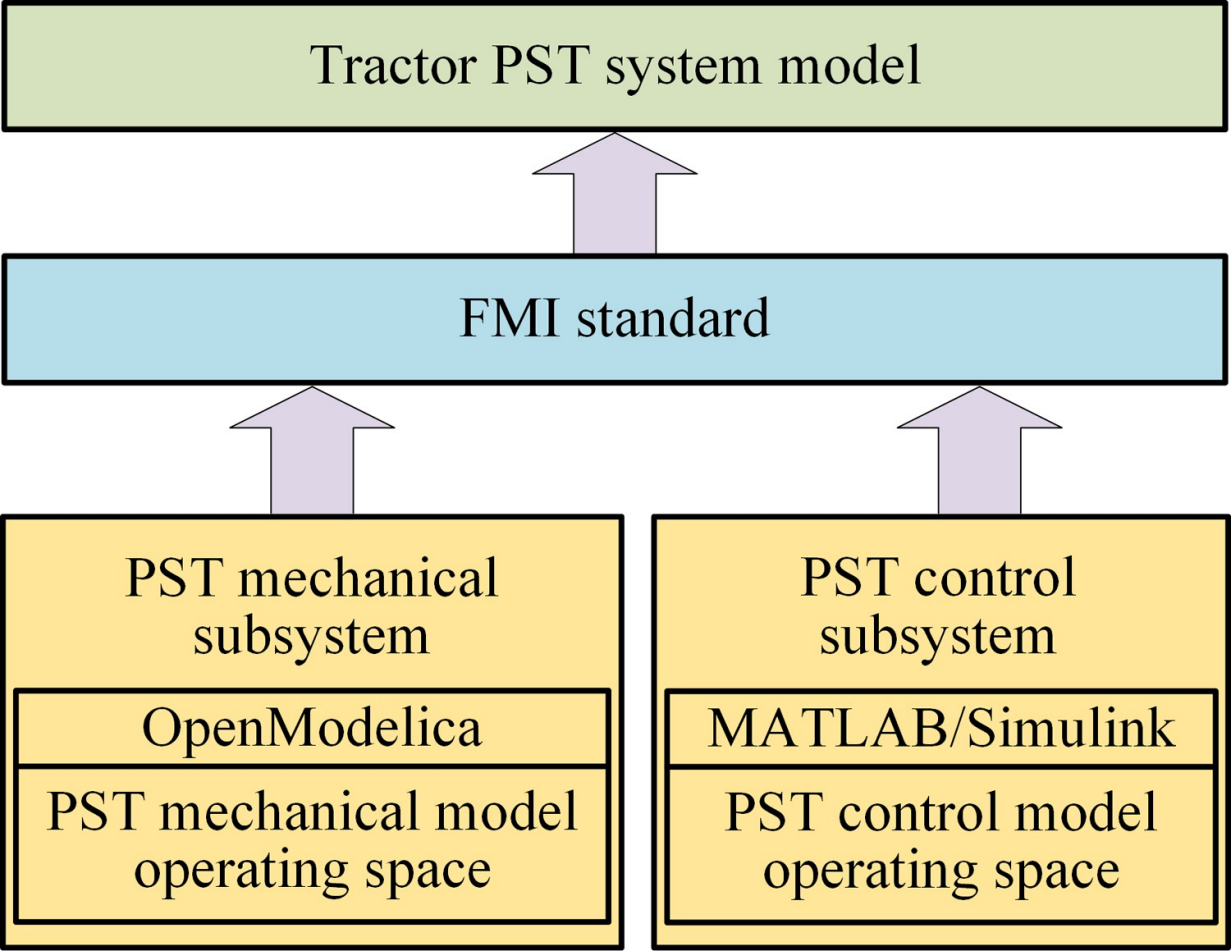
PST simulation system logical structure.

In this paper, the PST system co-simulation model is established based on FMI. Fig 2 shows the logical structure of the PST simulation system.

In Fig 2, the tractor PST system mainly includes mechanical and control subsystems, and simulation components mainly involve MATLAB/Simulink and OpenModelica software. The operating space of the PST mechanical model is the OpenModelica environment, which is an open source Modelica environment that supports the use of the Modelica language for modeling and enables efficient and large-scale FMI-based simulation [9]. The operating space of the PST control model is the MATLAB/Simulink environment. Based on the FMI universal interface standard, the PST mechanical subsystem model and the PST control subsystem model are integrated into the PST system model. The information interaction between the models, that is, the simulation component performs operations such as storing and taking out the input/output of the model, which is completed through the standard interface between the model and FMI.

## PST system model establishment

### PST mechanical system model

**Fig 3:**
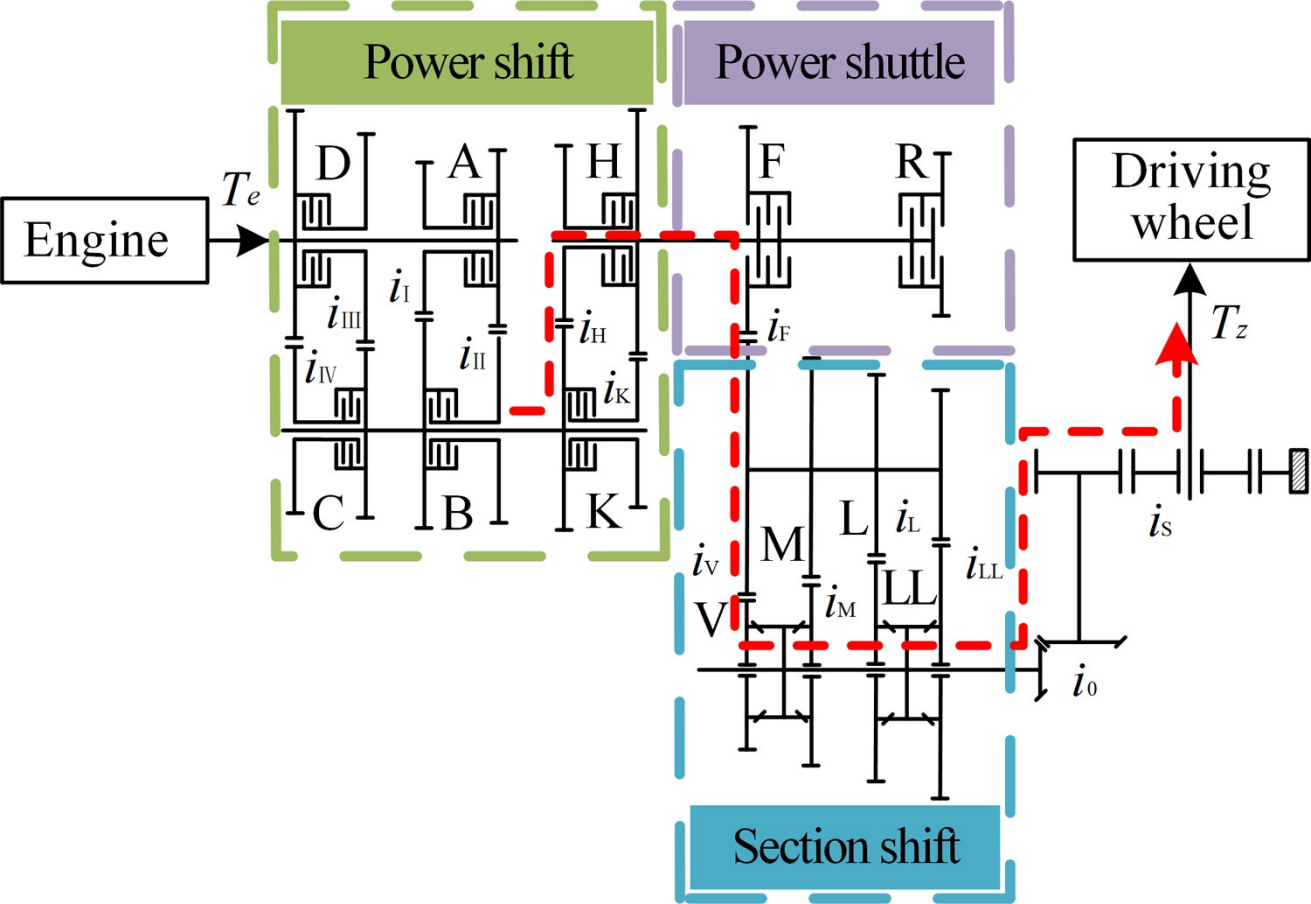
PST mechanical system power transmission diagram.

Fig 3 shows the power transmission of the tractor PST mechanical system. The power shift part includes four shift clutches (A, B, C, D) and high and low gear clutches (H, K), which can realize eight gear shifts. The power shuttle part includes forward and reverse clutches (F, R). The section shift part includes four section shift gears (LL, L, M, V). This paper adopts the dynamic analysis of the working conditions when the clutch H is engaged in the power shift part, the clutch F is engaged in the power shuttle part, and the synchronizer is engaged in the V section shift position in the section shift part. Power transmission route is shown by the red dashed line in Fig 3.

Table 1 shows the main parameters of the PST mechanical system.

**Table 1:** PST mechanical system parameters.

Parameter	Value
Transmission ratio	*i*_Ⅰ_ = 1.825
	*i*_Ⅱ_ = 1.556
	*i*_Ⅲ_ = 1.327
	*i*_Ⅳ_ = 1.132
	*i*_H_ = 1.455
	*i*_F_ = 1.180
	*i*_V_ = 0.390
	*i*_0_ = 6.300
	*i*_S_ = 6.000
Clutch friction plate number	A:6 B:7 C:6 D:6
Shaft moment of inertia	0.01
Friction coefficient	0.12

**Fig 4:**
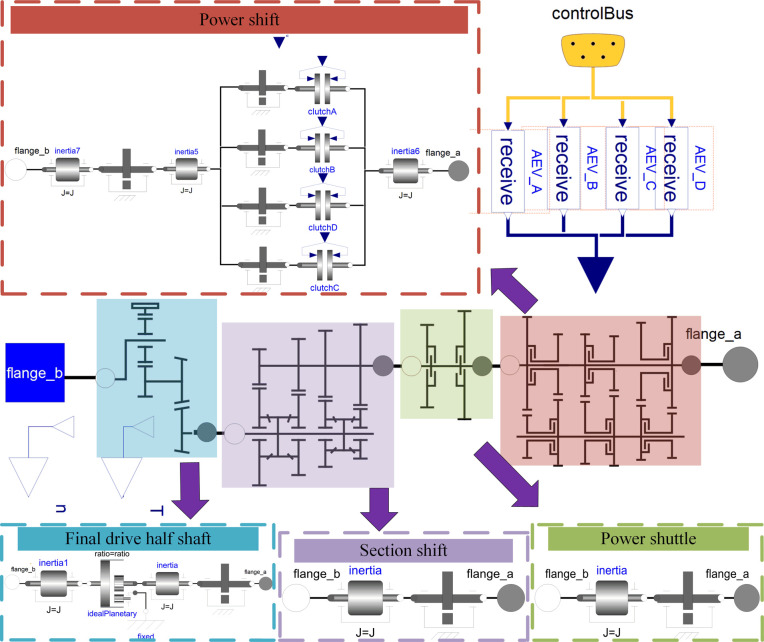
PST mechanical system model.

On the basis of these parameters, we established a mechanical system model in OpenModelica software and named it PST_mechanical_system, as shown in Fig 4. The mechanical system model includes power shift, power shuttle, section shift and final drive half shaft subsystem models. The input parameters are the engine speed and torque, and the PST gear signal, and the output parameters are the torque and speed of the PST system.

### PST control system model

Formulating the shift schedule is the basic problem that needs to be solved in the development of tractor PST control system. Under normal circumstances, tractors work in the field for a long time and require great power. Therefore, we formulate a three-parameter shift schedule including speed, slip rate and throttle opening. The tractor driving force is selected as the target control variable, the functional relationships between the tractor driving force and the vehicle speed, slip rate, and throttle opening are constructed, and finally the shift rules for upshift and downshift at different throttle openings and slip rates are obtained.

**Fig 5:**
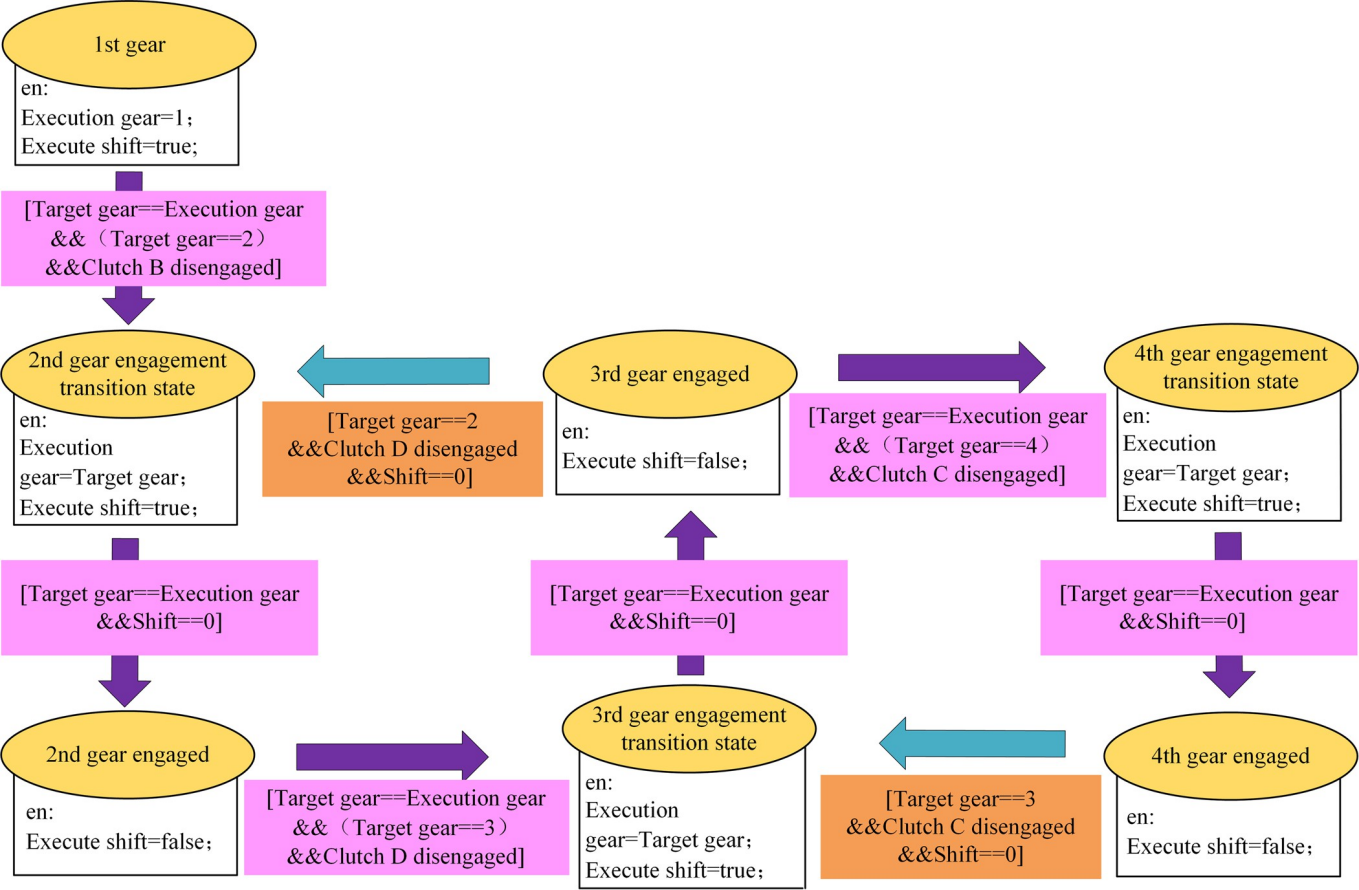
Shift logic judgment model.

The shift logic of power shift can be represented by MATLAB/Stateflow state machine, which we named ShiftLogic. Fig 5 shows the shift logic judgment model. The method for determining the target gear is as follows:

1. Input the throttle opening, slip rate and current gear signals into the shift schedule to obtain the critical vehicle speed for upshift and downshift;
2. Compare the current vehicle speed with the critical vehicle speed;
3. Output the gear shift signal for shifting if the gear shift conditions are met.

### PST co-simulation model

To realize the construction of the PST co-simulation model, we take MATLAB as the master pro-gram and OpenModelica as the slave program. Firstly, the PST mechanical system model needs to be generated as an FMU file, which can be achieved by setting the type of compiler, adding input and output parameters to the model, and selecting the corresponding FMI version and simulation mode. Then, import the FMU file into MATLAB/Simulink, which will automatically parse the XML file in it to obtain the model information, and call the solvers for simulation according to the same step length. In addition, it is necessary to establish the engine model and vehicle dynamic model for co-simulaiton, which are named Engine and VehicleDynamics respectively. Table 2 shows the main parameters of the engine and the tractor.

**Table 2:** The engine and tractor parameters.

Parameter	Unit	Value
Rated speed	rpm	2200
Calibrated torque	N·m	800
Rated power	kW	179
Torque reduction factor	-	0.03
Minimum use quality	kg	8600
Driving wheel radius	m	0.967
Total efficiency of mechanical transmission	-	0.9
Rolling resistance coefficient	-	0.07
Rotating mass conversion factor	-	1.2

**Fig 6:**
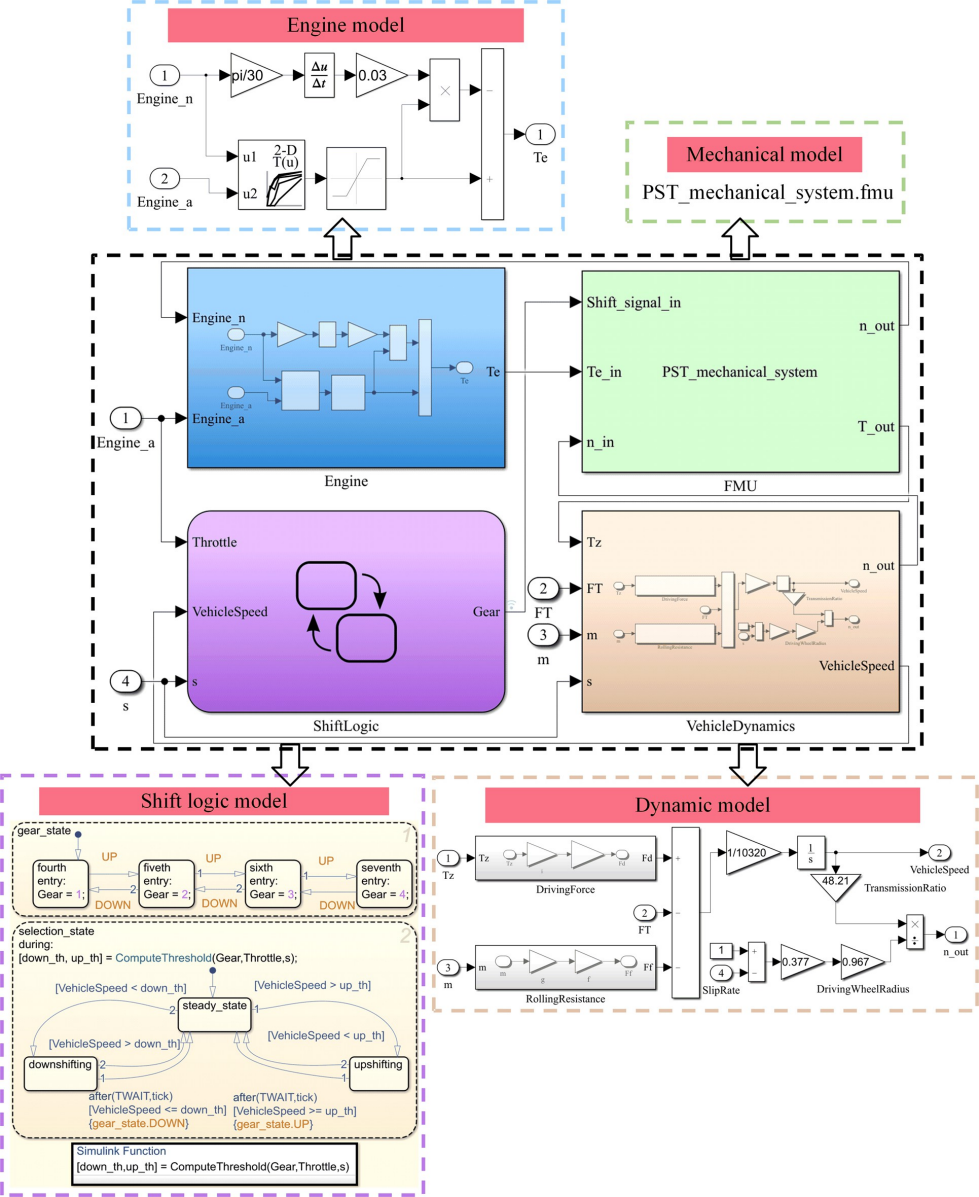
PST system co-simulation model.

According to the parameters in Table 2, the engine model and dynamic model are established in MATLAB/Simulink. Then, the mechanical system model, control system model, engine model, and dynamic model are integrated into the PST system co-simulation model through FMI, see Fig 6.

In Fig 6, the PST system co-simulation model mainly includes: the engine model (Engine), the shift logic model (ShiftLogic), the dynamic model (VehicleDynamics), and the mechanical model component FMU derived from OpenModelica. Its inputs are the gear signal (Shift_signal_in), torque (Te_in) and speed (n_in), and the outputs are speed (n_out) and torque (T_out).

## PST co-simulation model verification

Starting quality and shifting quality are important performances of the tractor PST system. The tractor starting speed, the torque and speed of the PST final drive half shaft are important basis for measuring the starting quality and shifting quality. Therefore, we verified the effectiveness of the PST system co-simulation model through a combination of simulation and test according to the tractor starting speed, the torque and speed of the PST final drive half shaft.

### Simulation and test

**Fig 7:**
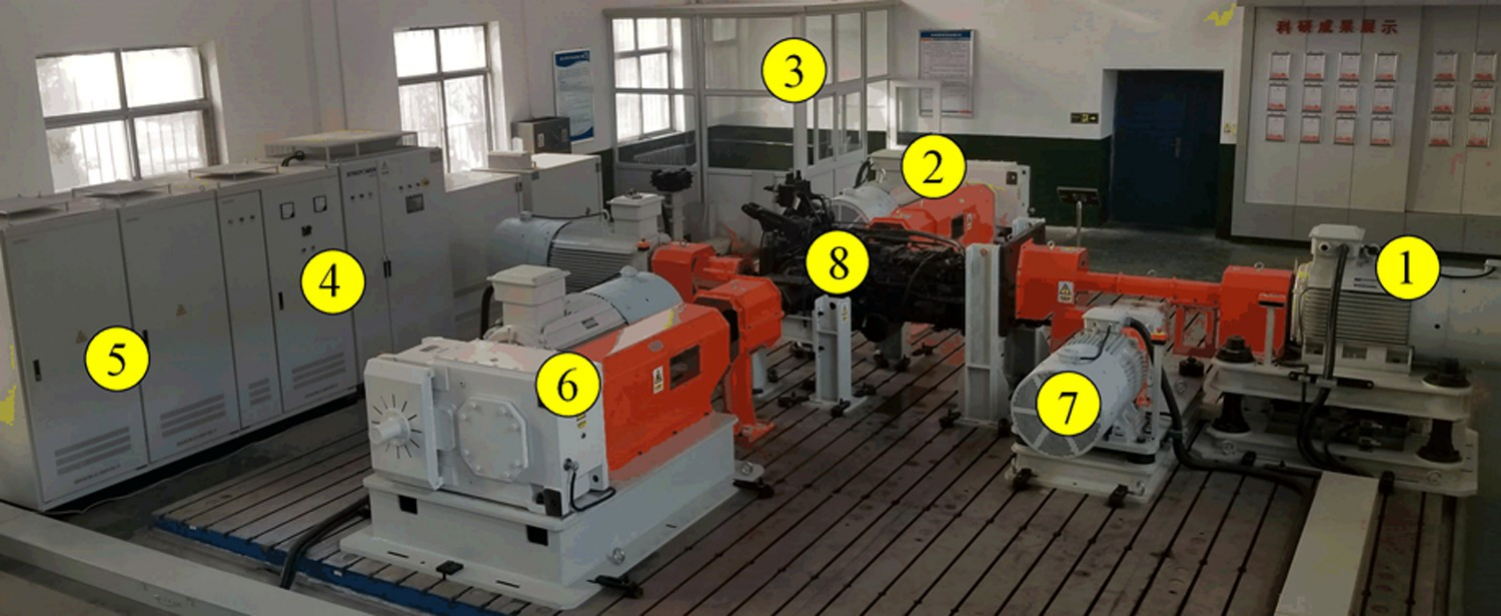
PST test bench. ① drive unit; ② left rear loading unit; ③ control room; ④ bidirectional power battery simulator; ⑤ inverter combination unit; ⑥ right rear loading unit; ⑦ front loading unit; ⑧ PST.

In the PST system co-simulation model, the throttle opening and slip rate are input for simulation. The PST test bench shown in Fig 7 is used for testing. The loading unit is connected to both sides of the PST drive shaft and the PST half shaft respectively. MATLAB/Simulink and PST test bench components are running in the control room. The PST test bench data acquisition controller collects PST test bench sensor signals and outputs PST test bench and PST shift actuator control signals.

The simulation and test conditions of the tractor starting speed and the PST final drive half shaft torque are set as follows: the starting gear is I gear, the input rotational speed is 2200 r/min, the input shaft load is 0.55 kN·m, and the driving shaft load is 0.75 kN·m. The tractor driving speed can be calculated by Eq (1):

V=(1−δ)πrn/30 (1)

where *δ* is the slip rate, which is 0.15; *r* is the driving wheel radius, which is 0.967m; *n* is the rotational speed of the PST output shaft.

The simulation and test conditions of PST final drive half shaft rotational speed are set as follows: the power shift transmission gear is upshifted from Ⅱ gear to Ⅲ gear and then downshifted to Ⅱ gear. The simulation outputs the gear signal when the gear is upshifted or downshifted through the control system, and the power shift signal is sent out through the shift handle button in the PST bench test.

### Result and discussion

**Fig 8:**
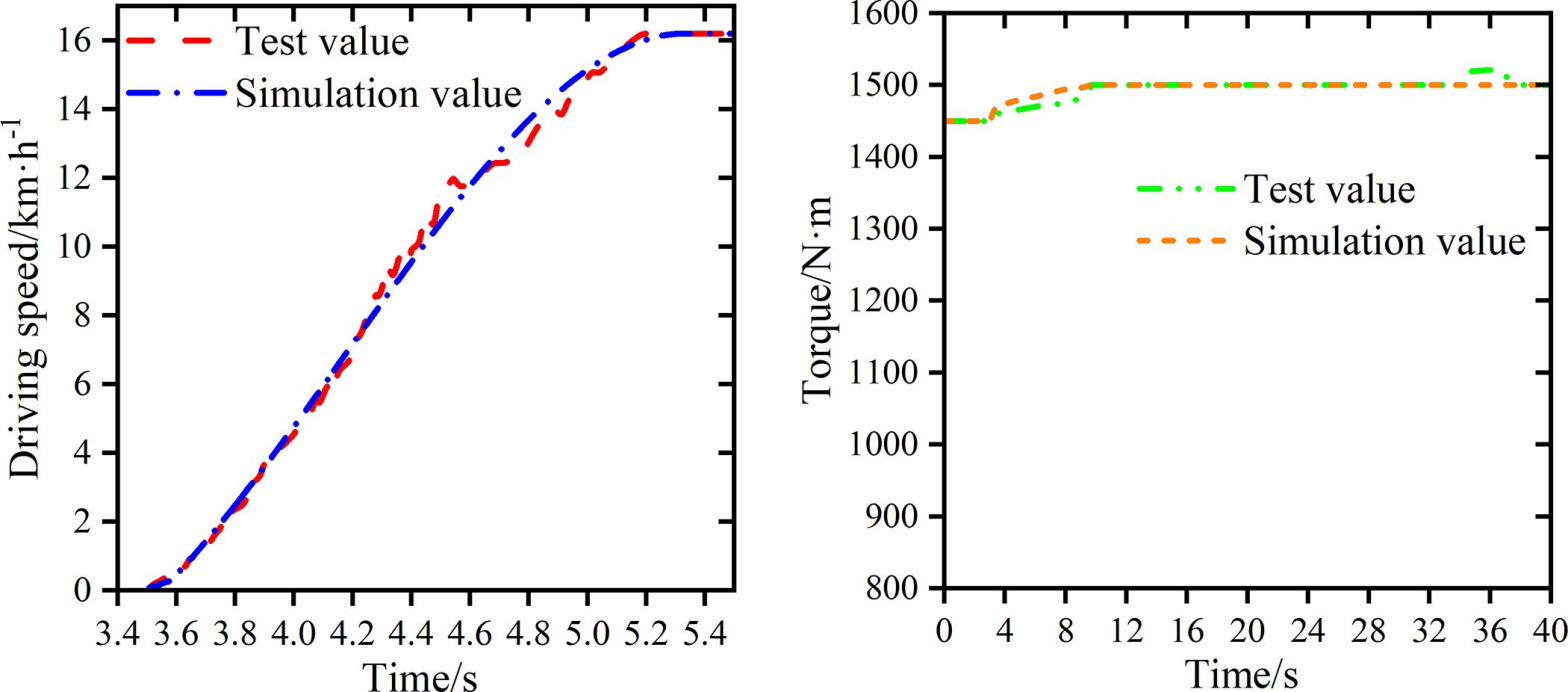
Simulation and test values of driving speed and final drive half shaft torque. (a) Driving speed (b) Final drive half shaft torque.

Fig 8 shows the comparison between the simulation and test results of the tractor driving speed, and the PST final drive half shaft torque when starting at the first gear with the high gear and light load. In Fig 8(A), the simulation start time is 2.7s, and the test start time is 2.8s. In the interval of 4.5s to 5s, the test driving speed fluctuates, which is caused by the elastic interference of the transmission shaft and gear meshing of the PST test bench. This is within a reasonable range and does not affect the accuracy of the test results. In Fig 8(B), the PST is steadily engaged in the first gear after 9s, and the final drive half shaft torque value of the simulation and test tends to the same value, which is 1.5kN·m.

**Fig 9:**
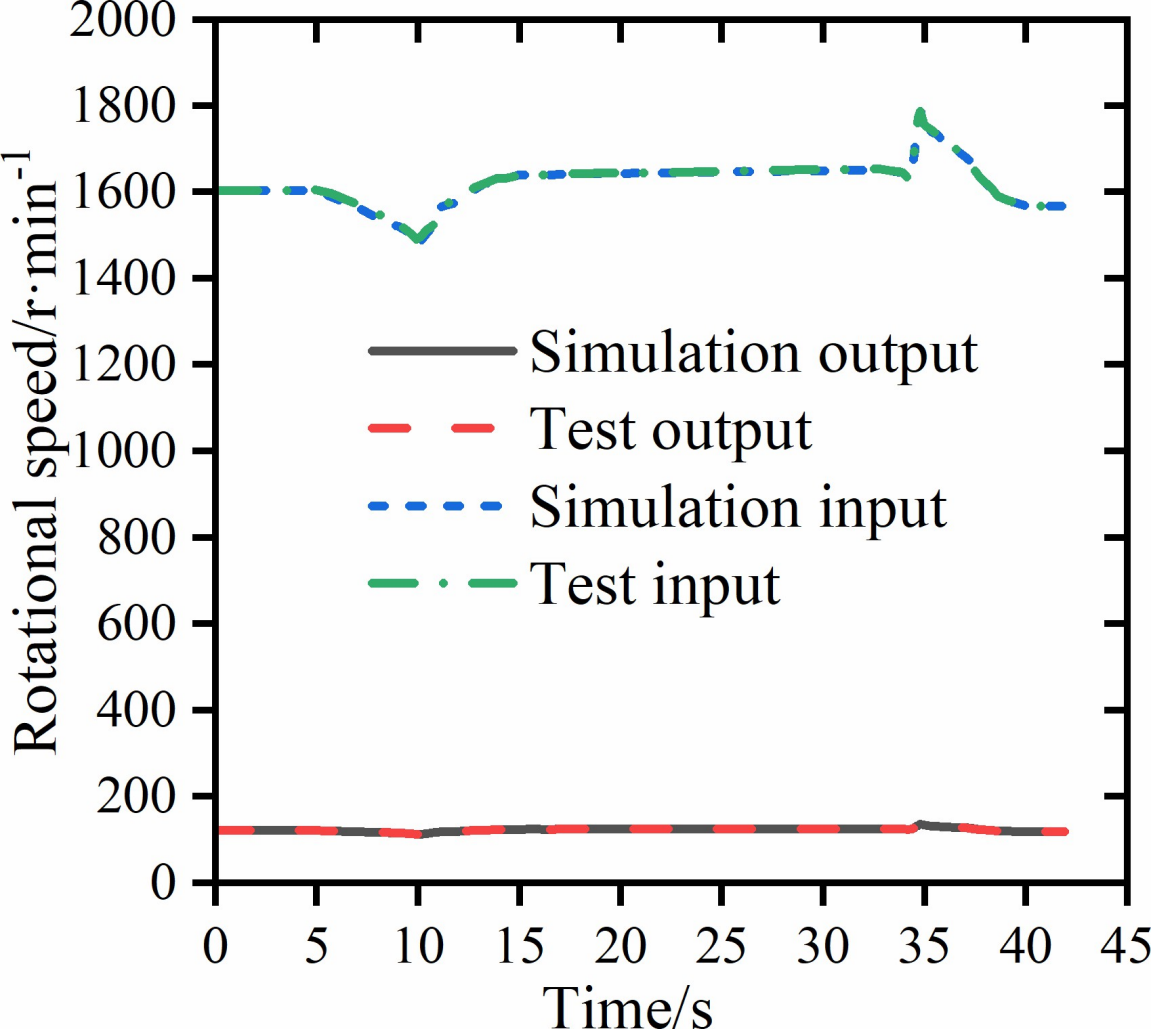
Final drive half shaft rotational speed simulation and test results.

Fig 9 shows the comparison of simulation and test results of PST final drive half shaft rotational speed. To check the validity of the simulation data, we can perform empirical mode decomposition on the simulation data and test data to form two sets of multi-order intrinsic mode function (IMF) and residuals, and calculate the Theil’s inequality coefficient (TIC) of the two sets of IMF components and residuals to perform a consistency check [36]. When TIC is less than 0.25, it can be considered that the simulation and test data are consistent. TIC value can be calculated by Eq (2):

$$TIC_{k}=\frac{\sqrt{\frac{1}{N}\sum_{i=1}^{N}{\left(IMF_{xi}^{k}-IMF_{yi}^{k+F-M}\right)}^{2}}}{\sqrt{\frac{1}{N}\sum_{i=1}^{N}{\left(IMF_{xi}^{k}\right)}^{2}}+\sqrt{\frac{1}{N}\sum_{i=1}^{N}{\left(IMF_{yi}^{k+F-M}\right)}^{2}}}$$ (2)

where *TIC*_*k*_ is the TIC value of the *kth* order IMF component of the simulation data and the *(k+F-M)th* order IMF component of the test data; *N* is the number of data time series points; $IMF_{xi}^{k}$ is the *kth* order IMF component of the simulation data IMF; $IMF_{yi}^{k+F-M}$ is the *(k+F-M)th* order IMF component of the test data IMF; *M* is the number of IMF decomposed by simulation data; *F* is the IMF number decomposed by test data, M≤F.

The simulation data and test data of the PST final drive half shaft speed are empirically decomposed. The simulation data obtains the 4th order IMF component, and the test data obtains the 5th order IMF component. The calculated four TIC values are 0.12, 0.13, 0.18, 0.12, respectively. The average TIC is 0.1375, which is less than 0.25, indicating that the simulation data is consistent with the test data. The speed changes slightly around the shift point, indicating that the PST did not appear to be power interrupted during the shifting process.

The simulation and test results of tractor starting speed, and the torque and rotational speed of the PST final drive shaft show that the simulation data matches the test data, proving the effectiveness of the PST co-simulation model and the feasibility of the PST co-simulation method based on FMI standard.

It can be seen from the research results that the way of using FMI-based co-simulation method combined with test verification method for tractor PST research is effective, which has a technical contribution to the development of tractor PST.

## Conclusions

In the present stage of tractor PST product development, multi-domain modeling and simulation are crucial tools. Through a mixture of modeling, simulation, and testing, this research explores and evaluates the tractor PST. Following are the key conclusions:

A method to solve the problem of tractor PST system co-simulation based on FMI standard is proposed, and the simulation mechanism based on FMI and the logical structure of PST simulation system are analyzed. The PST mechanical system model, control system model, engine and tractor dynamic model are established, and then the PST system co-simulation model is established based on FMI.

The simulation and test were carried out for the tractor starting speed, the PST final drive half shaft torque, and the PST final drive half shaft rotational speed. The results show that the simulation data is consistent with the test data, the established co-simulation model is accurate, and the proposed PST co-simulation method is feasible.

The PST co-simulation research carried out by this method can accurately and stably simulate the operation effect of a real tractor PST. Furthermore, the simulation object is not restricted to a fixed tractor PST model. Even if the demand changes, all that is required is to replace and modify the corresponding model, and then load different models to simulate and test them. As a result, it is reusable and extensible. PST developers can be liberated from the construction of the repetitive mechanical simulation environment in this way, which provides greater convenience for the design and iteration of the tractor PST, and improves the development efficiency of the PST system.

In summary, the method can significantly shorten the model development and maintenance cycle. It serves as an excellent guide for the digital design of the tractor PST as well as the development of the tractor as a whole. It generates new concepts for co-simulation research in the realm of agricultural machinery. In the future, models of the PST hydraulic system and other fields can be established and integrated on the basis of the method, and the PST simulation model can be gradually improved to enrich and complete the tractor transmission system design model library. The existing design and manufacturing technology can be further developed and integrated for use in the tractor product life cycle management process, allowing different professionals to test, analyze, and evaluate products from their own unique perspectives for remote collaborative development.

## Supporting information

S1 Data (XLSX)
